# Diversity, Encounter Rate and Detection of Non-Volant Nocturnal Mammals on Two Malaysian Islands

**DOI:** 10.21315/tlsr2024.35.1.4

**Published:** 2024-03-30

**Authors:** Priscillia Miard, Foo Kai Xin, Sapphire Hampshire, Nik Fadzly Nik Rosely, Henry Bernard, Nadine Ruppert

**Affiliations:** 1 School of Biological Sciences, Universiti Sains Malaysia, 11800 USM Pulau Pinang, Malaysia; 2 Institute for Tropical Biology and Conservation, Universiti Malaysia Sabah, Jalan UMS, 88400 Kota Kinabalu, Sabah; 3 Malaysian Primatological Society, 09000 Kulim, Kedah, Malaysia; 4 Faculty of Biology and Psychology, Göttingen University, Wilhelmsplatz 1, 37073 Göttingen, Germany

**Keywords:** Transects, Thermal Device, Red Light, Biodiversity, Detection, Transek, Peranti Haba, Lampu Merah, Biodiversiti, Pengesanan

## Abstract

Nocturnal mammals constitute a crucial component of tropical faunal diversity, but not much is known about the effects of anthropogenic disturbance on the habitat use and detectability of these species. We investigated which habitat and environmental variables impact the detectability of non-volant nocturnal arboreal mammals across varying habitat types at two tropical islands with different levels of anthropogenic development in Malaysia. We conducted night transect line and point count surveys following pre-existing paths in Penang Island and Langkawi Island between 2019 and 2020. We used a head torch with red filter and a thermal imaging device (FLIR) to enhance animal detection success. We calculated the encounter rates (individual km^−1^) for each species as a proxy for abundance. Overall, we detected 17 species, but did not find higher species diversity in intact forested environments compared to disturbed areas. Encounter rates of the most observed species were influenced by ‘time after sunset’ on the highly developed island of Penang, whereas on the rural island of Langkawi, detection was higher in sites with better canopy connectivity. Different species of non-volant nocturnal arboreal mammals use their respective habitats differently and thus, are differently impacted by varying levels of anthropogenic activities. Our results provided baseline data on the diversity, encounter rate, and detectability of these highly elusive species, which can also help to further improve methodologies for the detection of nocturnal wildlife.

## Abstract (ms)

Mamalia malam merupakan komponen penting dalam kepelbagaian fauna tropikal, tetapi tidak banyak yang diketahui tentang kesan gangguan antropogenik terhadap penggunaan habitat dan kebolehkesanan spesies ini. Kami menyiasat habitat dan pemboleh ubah persekitaran yang memberi kesan pengesanan mamalia arboreal malam yang tidak boleh terbang atau meluncur merentasi pelbagai jenis habitat di dua pulau tropika dengan tahap pembangunan antropogenik yang berbeza di Malaysia. Kami menjalankan tinjauan garisan transek malam dan kiraan titik mengikut laluan sedia ada di Pulau Pinang dan Pulau Langkawi antara tahun 2019–2020. Kami menggunakan lampu kepala dengan penapis merah dan peranti pengimejan terma (FLIR) untuk meningkatkan kejayaan pengesahan haiwan. Kami mengira kadar pertemuan (individu km^−1^) untuk setiap spesies sebagai proksi untuk kepadatan. Secara keseluruhan, kami mengesan 17 spesies, tetapi tidak menemui kepelbagian spesies yang lebih tinggi di persekitaran hutan yang utuh berbanding kawasan yang terganggu. Kadar pertemuan kebanyakan spesies yang diperhatikan dipengaruhi oleh ‘waktu selepas matahari terbenam’ di Pulau Pinang yang pesat membangun, manakala di kawasan luar bandar Pulau Langkawi, pengesanan lebih tinggi di tapak yang mempunyai sambungan kanopi yang lebih baik. Spesies mamalia arboreal malam yang tidak boleh terbang atau meuncur yang berbeza menggunakan habitat tersendiri secara berbeza dan oleh itu, dipengaruhi secara berbeza oleh pelbagai peringkat aktiviti antropogenik. Hasil kami memberikan data asas tentang kepelbagaian, kadar pertemuan, dan kebolehkesanan spesies yang sukar ditemui ini, yang mana juga boleh membantu untuk menambah baik metodologi untuk pengesanan hidupan liar malam.

**Highlights**

- A total of 17 nocturnal mammal species were detected and higher diversity was not correlated to a more intact habitat.
- In a more developed habitat, species were detected more often just after sunset whereas in a more rural area they were detected more in places with higher canopy connectivity. However, differences exist in terms of species detectability, which needs to be considered for future species-specific surveys.
- The use of red light coupled with thermal imaging should be standardised for nocturnal mammal surveys and white light should be avoided.

## INTRODUCTION

Nocturnal non-volant arboreal mammals are highly diverse and globally abundant and play an important role in their respective ecosystems, acting as pollinators (Carthew & Goldingay 1997; Winter & von Helversen 2001), seed dispersers (Hodgkison *et al*. 2003; Wells *et al*. 2009; Yasuda *et al*. 2009), pest control agents (e.g., against insects: Rode-Margono *et al*. 2014), or as food resources for predators (Akbar & Ariffin 1997; Hart 2007; Wiens & Zitzmann 1999). However, they are often notoriously difficult to study due to poor observation conditions at night and their elusive behaviour and cryptic appearance (Duckworth 1998; Silveira *et al*. 2003).

Nocturnal mammals are negatively affected by human activities, which can lead to overall biodiversity changes reducing ecosystem stability in the long-term (Achard 2002; Vitousek 1997). Major impacts of conversion of natural wildlife habitats for human use are local species extinction events (Vitousek 1997), reduced number of specialist small mammal species (Wells *et al*. 2007), loss of predators (Dirzo *et al*. 2014), and increased poaching rates of many, often already threatened, species due to easier access to dense forests in fragmented landscapes (Abernethy *et al*. 2013). Habitat fragmentation results in the creation of edge effects along ecotones that directly impact the distribution and dynamics of many species (Murcia 1995), including nocturnal, arboreal mammals which can also lead to their extinction in fragmented island habitats (Gibson *et al*. 2013). Edge effects and disturbance can increase, decrease or have minimal effect on the population density of a particular species (da Rosa *et al*. 2018) and thus, edge effects should be studied by individual species requirements and not by a group of species. For example, Umapathy and Kumar (2000) found that the density of flying squirrels increased when the habitat patch size was smaller and more disturbed.

Malaysia is home to at least 361 species of wild mammals (PERHILITAN 2017) with around 65% of them, not including bats, being nocturnal (Barret 1985). Several studies related to nocturnal non-volant mammal diversity have been conducted in Peninsular Malaysia (e.g., Kawanishi 1999; Othman 2000; Azlan & Sharma 2006; Azlan 2006; McShea *et al*. 2009; Bashir 2014; Ruppert *et al*. 2015; Sompud *et al*. 2016; Lo *et al*. 2018); however, there has not been focused research to assess the factors that may impact the diversity and distribution of nocturnal non-volant arboreal small mammal communities in different habitat types in islands of Peninsular Malaysia as it was explored for certain species of small mammals in Malaysian Borneo (Wells *et al*. 2004; Wells *et al*. 2014; Wearn *et al*. 2018).

These knowledge gaps are mainly due to the constraints of the research methods. Field survey techniques developed to study diurnal animals cannot simply be applied in the same way to study nocturnal arboreal mammals due to different behavioural ecology and detectability of the two guilds. Various techniques have been developed to assess distribution and abundance of nocturnal mammals in tropical rainforests (de Thoisy *et al*. 2008) based on study species, cost, resource limitations, and environmental conditions (Silveira *et al*. 2003). These methods include live-trapping, radiotelemetry or GPS tracking, camera trapping, spotlighting, census/transect walks, direct/opportunistic observations (Catling *et al*. 1997; McComb *et al*. 2010), but a systematic assessment of the efficiency to detect nocturnal animals is often lacking, obscuring the factors that determine the presence or habitat selection of a species.

The aim of the study was to better understand the factors that impact the diversity and habitat selection of non-volant nocturnal arboreal mammals, especially species vulnerable to anthropogenic land-use changes, on two Malaysian islands with different anthropogenic impact and development, i.e., Langkawi and Penang.

To achieve the aim of the study, we used a two-fold approach: firstly, we collected point location and transect presence data for each species along transects at a study site to calculate: (a) species diversity indices; (b) encounter rates; and (c) to map the encountered species across habitat types with varying anthropogenic impact. Secondly, we investigated which method-specific and/or site-specific factors influence the detection probability of species, thus, providing vital information on how to reduce detection bias and improve nocturnal wildlife survey methods.

We anticipated lower species diversity in highly disturbed habitats compared to undisturbed or slightly disturbed habitats (Dornelas 2010). Therefore, we hypothesised that site-specific factors, such as anthropogenic disturbance level and lack of canopy cover may negatively impact species encounter rates (Nekaris *et al*. 2014).

As imperfect detectability is unavoidable in most cases, it is important to understand which specific environmental or disturbance factors may affect detection probability of a species in order to improve animal detection rates (Buckland *et al*. 2004; Einoder *et al*. 2018). We predicted that environmental factors including aspects of habitat quality (vegetation), weather conditions and topography, as well as anthropogenic factors including human settlements and their activities, would influence the detectability and/or distribution of the non-volant nocturnal arboreal mammal species (Nekaris *et al*. 2014; Rode-Margono *et al*. 2014; Buckland *et al*. 2015).

## METHODS

### Study Sites

**Figure 1:**
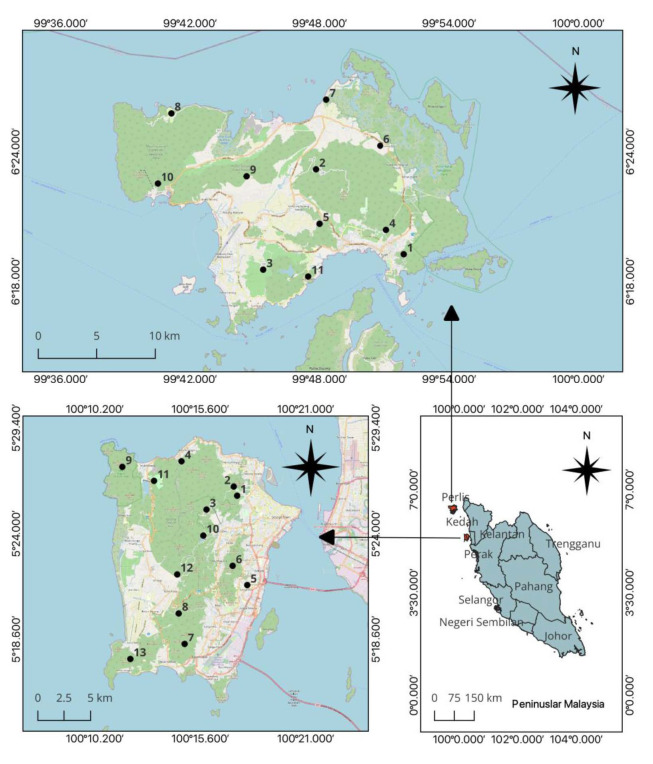
Locations of the study sites (bottom right) and map of Penang Island (bottom left) and Langkawi Island (top) with locations of survey sites. (Source: Priscillia Miard created with QGIS, Open Street Map Source) *Description of the sites:* **Penang Island:** 1. Youth Park, 2. Penang Botanic Gardens and 11. Taman Rimba Teluk Bahang (recreation park with dipterocarp forest), 3. Penang Hill (eco-tourism attraction surrounded by hill dipterocarp forest, vegetable farms and orchards). 4. Batu Feringghi (human settlements near coast, fringed by lowland dipterocarp forest), 5. USM (main campus of Universiti Sains Malaysia), 6. Bukit Gambir (dipterocarp forest), 7. Bayan Lepas south and 8. Bayan Lepas north (orchards and plantations near human habitat), 9. Penang National Park (protected coastal, hill/lowland dipterocarp forests and mangrove forest areas), 10. Ayer Hitam (orchards and dipterocarp forest), 12. Balik Pulau (orchards and village), 13. Gertak Sanggul (orchards and dipterocarp forest). **Langkawi Island:** 1. Penarak (village, park and dipterocarp forest), 2. Gunung Raya (dipterocarp forest), 3. Bukit Lembu (village, plantations and dipterocarp forest), 4. Sungai Tarom (village and dipterocarp forest), 5. Padang Gaong (villages and rubber plantations), 6. Kilim (village near dipterocarp forest), 7. Tanjung Rhu (mangrove and coastal beach), 8. Golf Course (open greenery and disturbed dipterocarp forest), 9. Kuala Teriang (village and orchards), 10. Cable Car (recreation park with dipterocarp forest), 11. Bukit Malut (orchards and plantations).

We conducted this study on the island of Penang (5°22′ 2″N, 100°14′ 55″E) and the main island of Langkawi (6°21′ 0″N, 99°48′ 0″E), both at the West coast of Peninsular Malaysia (Fig. 1). Although, the Langkawi archipelago with its 99 islands is bigger in area size than Penang, its main island (320 km^2^) is similar to Penang Island (293 km^2^). We selected these two islands because of their close geographic proximity, similar area size and because both have different anthropogenic development, with Penang being highly developed and urban compared to the more rural and less developed Langkawi. Both islands have high abundance of some nocturnal mammal species, probably due to a lower predation rates than on the mainland (Meijaard 2003; Luna-Jorquera *et al*. 2012).

Penang Island is one of the most developed states in Malaysia hosting its second largest city (Georgetown) in terms of population density (722,384 habitants; population density of 2,465.47/km^2^) (Malayan Nature Society 1999; Department of Statistics Malaysia 2010; Deuskar *et al*. 2015). The central part of the island consists of lowland tropical rainforest and hill forest recognised as Penang Hill Biosphere Reserve by UNESCO; however, the landscape of Penang has been experiencing quick and significant habitat changes to meet the requirements of the growing population over the last three decades (Masum *et al*. 2017). Much of the island has now been converted for urban and agricultural use (Deuskar *et al*. 2015; Weng Chan 1998). The distribution and abundance of nocturnal mammals, such as bats (Shafie 2016; Huang *et al*. 2019; Lim *et al*. 2019) and small terrestrial rodents, mainly Muridae (Mohd Sah *et al*. 2006a; 2006b), have been fairly-well studied in Penang Island, but other nocturnal mammals, such as pangolins, mouse deer, porcupines, slow lorises, colugos, and flying squirrels, are yet to be comprehensively assessed here.

Langkawi is an archipelago of 99 islands and the population of the main island is comprised of 65,000 people (Department of Statistics Malaysia 2010). Langkawi is recognized as a UNESCO Geopark due to its unique karst landscape formed during multiple geological events (Leman *et al*. 2007; 2008). Mammal surveys on the island are limited to small mammal trapping studies for rats, squirrels, treeshrews, and bats (Lit *et al*. 2011; Nor *et al*. 2007). Other nocturnal mammals, such as the Malay civet, small-toothed palm civet, mouse deer, and colugos are reported from the island, but no specific surveys on these species have been conducted here (Lit *et al*. 2011; Meijaard 2003).

### Night Transect Survey Method

On each island we determined survey sites with similar habitat parameters where we conducted night transect walks. We used Open Street Map and QGIS to identify suitable survey sites on the desktop prior to going to the field to ensure that a minimum of seven distinct 500 m-long transect paths were available in each site following Buckland *et al*. (2015) (also see Buckland *et al*. 2004; Marshall *et al*. 2008).

Survey sites were chosen on both islands to encompass areas of varying altitude, habitat type, and anthropogenic disturbance, where each site had between 9 to 19 distinct transects. We sampled one site over multiple nights, but each transect was surveyed only once (Star *et al*. 2011; Marsden *et al*. 2016) (Table 1).

We recorded data in Penang Island from 19 March 2019 until 30 September 2019, and in Langkawi Island from 3 October 2019 until 2 February 2020. We surveyed transects with two observers simultaneously between 1900 h and 0200 h on each survey night, mostly during clear nights without heavy rain to achieve similar probabilities for animal detection and for the safety of the observers.

Sites and transects were selected opportunistically, due to terrain, accessibility, and the required availability of the 500 m transects which correlates to area size in one habitat type (Nekaris *et al*. 2008; Marsden *et al*. 2016). We used existing paths along established trails and roads to minimise disturbance for the animals (Nekaris *et al*. 2008; McComb *et al*. 2010). Existing paths are easier to walk silently, especially in dense vegetation and complex terrain, such as a hilly rainforest, compared to linear transects that need to be established and artificially “cut” through the forest and are harder to follow in a natural environment (Marshall *et al*. 2008; Plumptre *et al*. 2013). Existing trails also offer better visibility to spot animals as the vegetation to the canopy is already opened to some extent (Duckworth 1998; Kawanishi 1999).

We recorded all animal observations made while slowly walking along the transects using a headtorch (Clulite HL13 with red filter) to detect eyeshine as well as during 5-min stops at every 100 m along the transects to conduct point counts using a thermal imaging device (FLIR Scout III model 640 monocular, FLIR, USA). Focardi *et al*. (2001) showed that thermal imaging yields better detection results compared to conventional spotlighting methods for certain species (e.g., wild boar). The FLIR model used in this survey can detect a human up to 1,140 m in open landscape and has a thermal sensitivity of < 50 mK at f/1.0. It has a field of view of 18° × 14° NTSC and a 33 mm fixed focus focal length with a 2× and 4× time zoom (FLIR, https://www.flir.com/products/scout-iii/?model=431-0019-31-00&vertical=public+safety&segment=solutions). The detector is a 640 × 512 VOx Microbolometer and can detect waveband between 7.5–13.5 μm. It can operate at temperatures between −20°C to 50°C and it is waterproof (FLIR 2022).

The head torch we used was equipped with a red-light filter, which is recommended for surveying nocturnal mammals, as their densely packed rods cells are insensitive to wavelengths longer than 650 nm (i.e., deep red colour; Bowmaker 2008). Therefore, red light is less disturbing and minimises the awareness about the observer’s presence increasing the time of direct animal observations (Southern *et al*. 1946; Vestal & Hill 1972).

Animals can also be detected by their sounds when they call, move in trees or on the ground, or by droppings of fruits during feeding events along the transect (Catling *et al*. 1997; Duckworth 1998). For this study, we only recorded calls made by species that we could identify.

Whenever we spotted animals along the transect, we took a picture with a camera (Nikon model D3100; 55–300 mm lens, Nikon Corporation, Japan) to confirm the species when required (identification followed Francis [2019]). The camera flash was never directed towards the eyes of the animals to avoid disturbance (Glen *et al*. 2013; Henrich *et al*. 2020). We noted species, GPS location, number of individuals (if group-living), estimated distance to the observer, estimated height of animal above forest floor/in the tree, position on the tree (i.e., on branch, on stem, above canopy), and behaviour at first encounter. We also recorded variables including weather (i.e., dry, rainy, cloudy windy), canopy cover and connectivity, type of habitat surveyed, and type of path used (see Table 2 for detailed description).

### Canopy Cover Indices

We used QGIS software (version 3.8) and Google Maps satellite images (2020) to classify land use type and created a canopy cover index (CI) for each survey site.

We classified the land use in the satellite images into (a) *forest* (i.e., old growth and secondary forests with intact canopy cover); (b) *agriculture 1* (i.e., mixed tree orchards with intact canopy cover, e.g., durian *Durio zibethinus*); and (c) *agriculture 2* (i.e., monocultures such as oil palm *Elaeis guineensis*, coconut palm *Cocos nucifera*, and rubber *Hevea brasiliensis*, with disturbed canopy cover), *water surface*, and *human settlements* (without canopy cover). Following these classifications, we created the CI for each surveyed site as the percentage of *forest* and/or *agriculture 1* against the total polygon size.

We created a minimum convex hull polygon containing all survey transects in a site to determine the total surveyed habitat patch size. We performed supervised land use classification (i.e., land cover classes assigned manually by the user) as the size of each survey site was too small for automated classification (size of the convex hulls: 0.72 km^2^–5.77 km^2^; Jacobson *et al*. 2015; Phillips *et al*. 2017).

We removed *water surface* from the total area calculation as we focused on species that do not use this habitat type and as it does not necessarily indicate anthropogenic disturbance.

### Data Analysis

We used RStudio (2023.03.0+386) for all analyses.

The data for this survey was analysed by two formats: encounter rate (ind. km^−1^) and encounter/no encounter (0/1) for the Generalised Linear Mixed Model (GLMM) analysis looking at detection.

#### Species encounter rates

We calculated the species encounter rates with their standard error as the number of detected individuals per kilometer (ind. km^−1^) following Sutherland (2002) (i.e., number of individuals per unit area; Nekaris *et al*. 2008; Rinehart *et al*. 2014).

#### Species diversity

We calculated four alpha-diversity indices (Whittaker 1977) and compared them between sites using the *Vegan* package in R (*v2.5.6;* Oksanen *et al*. 2019): Shannon-Wiener Index (H’), Simpson Index (1-D), species richness (S; the number of different species occurring at a site), and Pielou’s evenness (J’; the distribution of number of individuals in a species) in a given community (Morris *et al*. 2014).

#### Species accumulation curves

We created species accumulation curves for each survey site on both islands using the *Vegan* package (*v2.5.6*, Oksanen *et al*. 2019), with 100 random reorderings to confirm if the number of sites surveyed was sufficient to detect all present species in an area, and how many survey sites would be required for each species to maximise detection success. These accumulation curves for both islands confirmed that different species need different survey efforts, but also that required number of sites differed for the same species between both islands (see Appendix).

On Penang Island, all but two species reached an asymptote after 13 sites surveyed, and on Langkawi Island, all but five species reached an asymptote after 11 sites surveyed.

#### Factors affecting nocturnal mammal detections

We ran a GLMM using the *lme4* package (Bates *et al*. 2015). Data exploration was carried out following the protocol described in Zuur *et al*. (2010). Due to collinearity, we removed three variables from the final analysis: *habitat patch size* (correlated with vegetation patch size), *canopy cover index* (%) (correlated with disturbance), and *rain* (correlated with cloud cover).

We used a binomial error structure and the logit link function to test which variables affected animal detection (McCullagh & Nelder 2019) by modelling their actual presence data (see Appendix). Animal sightings for this analysis were coded as encountered (1) or not encountered (0) along a transect as the smallest independent sampling unit. For constructing the GLMM, only non-volant nocturnal mammal species were included. Model selection was done by separating method-specific and site-specific factors into two models with all predictors or explanatory variables inserted in the models as well as each variables independently.

We further examined a set of models with all possible combinations of the explanatory variables and ranked them by the Akaike Information Criterion (AIC) to find the best models (Delta AIC < 2). For analysing method efficiency, we conducted analysis by pooling all species, by species individually, and separated by study area (Penang vs. Langkawi).

## RESULTS

On Penang Island, we surveyed a total of 13 survey sites with 187 transects covering 93.5 km of transect length at altitudes of 0 m a.s.l. to 782 m a.s.l. On Langkawi Island, we surveyed 11 survey sites with 161 transects covering 80.5 km of transect length at altitude between 0 m a.s.l. and 604 m a.s.l. We had a total survey area of 33.25 km^2^ on Penang Island and 19.80 km^2^ on Langkawi Island.

### Encounter Rates of Non-Volant Nocturnal Arboreal Mammals

In Penang, a total of 330 encounters of nocturnal arboreal non-volant mammals belonging to 12 species of nine families were recorded at the survey sites (Table 3). In Langkawi, we recorded a total of 225 encounters of 11 species and 8 families (Table 3).

Two species were only encountered on one island: red-checked flying squirrel, *(Hylopetes spadiceus)* was found at a low encounter rate (0.20 ± 0.7 ind. km^−1^) on Langkawi. Horsfield’s flying squirrel (*Iomys horsfieldii)* was found at a high encounter rate (1.43 ± 2.0 ind. km^−1^) in Penang. Sunda slow loris (*Nycticebus coucang)* was encountered more in Langkawi (0.29 ± 0.6 ind. km^−1^) than in Penang (0.12 ± 0.4 ind. km^−1^), while the common palm civet (*Paradoxurus hermaphroditus)* was encountered more often in Penang (0.41 ± 0.5 ind. km^−1^) than in Langkawi (0.29 ± 0.4 ind. km^−1^; Table 3).

### Species Diversity

Species diversity of each site is presented in Tables 4 and 5. In Penang, the survey site with the lowest canopy cover (i.e., USM; CI = 20.48%) was also the site with the lowest species richness (S = 1) and the least number of sighted individuals (*n* = 2) (Table 4). However, this was not the case in Langkawi, where the survey site with the lowest canopy cover was Penarak (CI = 61.26%), but the site with the lowest species richness (S = 1) and lowest number of sighted individuals (*n* = 2) was Tanjung Rhu (CI = 78.38%) (Table 5).

The site with the highest canopy cover in Penang (i.e., National Park; CI = 99.53%) was not the site with the highest number of individuals (*n* = 29) or highest species richness (S = 6). The site with the highest number of sighted individuals (*n* = 38) was Bayan Lepas North (CI = 73.42%), and the sites with the highest species richness (S = 7) were Batu Ferringhi (CI = 87.47%) and Taman Rimba (CI = 96.61%). The other biodiversity indices showed similar results for all survey sites (Table 5). The results for Langkawi were similar, where the site with the highest canopy cover (Gunung Raya; CI = 98.52%) was not the site with the highest number of sighted individuals (n = 36) (i.e., Golf Course; CI = 68.73%) or the highest species richness (S = 8) (Cable Car; CI = 90.74%).

### Factors Affecting Nocturnal Mammals Detections

For both islands, different factors impacted the detection of nocturnal mammal species (Table 6).

For all the species pooled together, *the time of sighting after sunset* (coefficient: −3.513; wt = 0.517) negatively influenced their detection, with the closer the time to midnight the fewer animals detected on Penang Island, whereas on Langkawi Island, it was a higher *canopy connectivity* (coefficient: 0.691; wt = 0.317), that positively influenced their detection.

*Galeopterus variegatus* detection on Penang Island was influenced by the following factors (wt = 0.398): *Path type* (Forest road: 16.699, Forest trail: 16.159, Village road: 14.956) and *wind* (0.646) positively influenced detection with more animals sighted on all path used except big roads or during windy nights, whereas more cloud cover (−0.457), higher ambient temperature (−0.038) and closer to midnight the less likely the detection of an animal (−1.025).

On Langkawi Island, all factors included in the site-specific model impacted detection (wt = 0.618). Detection was higher in parks (0.260) and villages (0.998) and lower in orchards (−0.581) and plantations (−0.628) on Langkawi Island. *Altitude* (−0.003) and vegetation *patch size* (−0.364) negatively influenced detection, i.e., animal detection was lower at higher altitude and larger patch size, whereas *canopy connectivity* (0.831), *distance to road* (0.00002), *distance to human settlements* (0.0004) and *disturbance* (0.003) positively influence it, i.e., habitat quality affect detection, and the further away we are from anthropogenic activities, the greater the detectability.

*Iomys horsfieldii* detection on Penang Island was impacted by most method-specific factors (wt = 0.415) except for *cloud* and *moon*. *Path type* (Forest road: 0.765, Forest trail: 1.616, Village road: 1.742) and *temperature* (0.032) positively influenced detection, whereas *wind* (−0.628), *humidity* (−0.051) and *time after sunset* (–1.205) negatively influenced detection.

*Nycticebus coucang* detection (wt = 0.223) was positively impacted by *cloud* (0.950) and negatively impacted by *humidity* (−0.089) and *time after sunset* (−0.915) on Penang Island, but only negatively impacted by *disturbance* (coefficient: −0.057; wt = 0.350) on Langkawi Island.

*Paradoxurus hermaphroditus* detection was negatively impacted by the *time after sunset* (coefficient: −0.8685, wt = 0.520) on Penang Island, but positively on Langkawi Island (0.488). On Langkawi Island the *wind* (−0.689) negatively impacted detection (wt = 0.165) as well.

*Petaurista petaurista* detection was negatively impacted by *disturbance* (−0.234) and *distance to road* (−0.005) on Penang Island (wt = 0.281), and positively influenced by *path type* (Forest road: 17.851, Forest trail: 15.902, Village road: 16.056) and *time after sunset* (0.621) on Langkawi Island (wt = 0.432).

## DISCUSSION

### Species Encounter Rates

The most frequently encountered species in Penang were *Galeopterus variegatus* and *Iomys horsfieldii*. Both species were present in 12 out of 13 survey areas, except for the university campus. For these gliding mammals, trees must be spaced at a minimum distance of 5m for successful gliding and landing (Ando & Shiraishi 1993; Vernes 2001; Nasir 2013). At the campus, numerous large trees are present but mainly on species is present, *Samanea saman*, and planted resulting in original tree species locally extinct as well as mammal species or not providing sufficient food availability even if some local fruit trees are present such as mango trees (Tahir *et al*. 2020). Streetlights along the campus roadsides may cause light pollution and increase the likelihood that nocturnal mammals are detected by predators (e.g., owls that are abundant on campus) or caught by humans (Beier 2006). Therefore, the campus, which was the most developed survey site in Penang, seems not suitable for most forest-dwelling nocturnal mammals, especially colugos and flying squirrels.

According to Medway (1969), there are five species of flying squirrels in Penang Island: the large black flying squirrel (*Aeromys tephromelas*), Horsfield’s flying squirrels (*Iomys horsfieldii*), red giant flying squirrel (*Petaurista petaurista*), whiskered flying squirrel (*Petinomys genibarbis*), and smoky flying squirrel (*Pteromyscus pulverulentus*). However, we only encountered two species, *I. horsfieldii* and *P. petaurista*, which indicates that the other species that once occurred on the island are either present in very small populations and not detected in this study or may have already become locally extinct. Most encountered squirrels immediately escaped from the light of the headtorch and hid in dense vegetation when spotted. *Iomys horsfieldii* is a small, fast moving flying squirrel that is widespread in forests and plantations at all elevations (Aplin & Lunde 2016). We detected them by visual sightings and their specific calls, but although this species is quite common, only very few studies have been conducted on them.

The most frequently encountered species in Langkawi was also *Galeopterus variegatus*. Although colugos can be considered common in Malaysia, they do not receive attention from researchers or the public. This study confirms that they are common on both islands and present at almost all sites with more than one individual. Colugos in Langkawi were encountered in ten out of 11 study sites, except for Tanjung Rhu. This site is located between a beach and a mangrove forest and differs in its vegetation from the other sites as the dominant tree species here is the Australian pine tree (*Casuarina equisetifolia*). This tree species seems unsuitable for most nocturnal arboreal mammals, as only one species was sighted here, *Parodoxurus hermaphroditus*.

Twelve of the 14 sighted nocturnal mammal species in this study are categorised as “Totally Protected” with exception of *Tragulus kanchil and Hystrix brachyura*, which are listed as “Protected” species that can be hunted by indigenous communities (Wildlife Conservation Act 2010). Currently, the IUCN conservation status of *Nycticebus coucang* is Endangered (Nekaris *et al*. 2020), and *Hemigalus derbyanus* is categorised as Near Threatened (Ross *et al*. 2015), while the other encountered species are listed as Least Concern (Aplin 2017; Aplin & Lunde 2016; Boeadi & Steinmetz 2008; Duckworth *et al*. 2016; Duckworth 2016; Timmins & Duckworth 2015).

### Species Diversity of Each Survey Site

A low canopy cover percentage did not translate into a lower number of sighted individuals nor lowest species richness at a survey site, which contradicts our predictions. Often, a more diverse habitat directly translates into a more diverse species composition, thus, higher species richness (Luna-Jorquera *et al*. 2012). However, some species can adapt to a certain level of anthropogenic disturbance due to increased food availability along forest edges, especially near agricultural land (Laurance 1991; Luna-Jorquera *et al*. 2012). According to the “intermediate disturbance hypothesis” (Connell 1978; Dornelas 2010), intermediate levels of disturbance may promote higher levels of diversity due to sufficient time between disturbance for many species to colonise, which however is not long enough for competitive exclusion. Meanwhile, low levels of disturbance allow high competition to reduce diversity, while elevated levels of disturbance reduce diversity by only allowing most resistant species to survive (Dornelas 2010). This would confirm the presence of most species within areas of a medium level of disturbance on both islands. However, this could also be explained by the fact that animals are easier to detect at sites with lower canopy cover indicating a detection bias in highly dense forests.

### Factors Affecting Nocturnal Mammal Detections

#### All species pooled together

When studying nocturnal mammals, low detection rate is common as they do live in smaller groups unit or solitary compared to diurnal species (Nekaris *et al*. 2008; Pereira *et al*. 2017; Rocha *et al*. 2021).

Animal detection was impacted differently on both islands, but also for different species, and this is corroborated by other studies as different species and sites have different characteristics to account for, such as rarity, behaviour, but also habitat density, suitability, and disturbance (Bailey *et al*. 2004; Guillera-Arroita 2017). These results can help us to better understand the effectiveness of our method pertaining to survey effort, observer skill, and detection device, and adjust future studies.

Our results are in accordance with the hypothesis that human disturbance affects species distribution (Sala *et al*. 2000; Wilson 2002) as the availability of suitable habitat is highly correlated to human activity, but suitability often depends on species-specific factors correlated to dietary needs and behavioural plasticity (Crane *et al*. 2014; Santini *et al*. 2019; Bista *et al*. 2022). The behaviour and ecological importance of many species detected in this study are not fully understood yet, thus, information on the factors potentially influencing their detection can give us better insights into how they use of their habitat.

On Penang Island, we found that the *time of survey* was the most important factor in predicting species detection. This indicates a general behavioural trait of arboreal species with a peak feeding and foraging time just after waking up as shown from studies on the activity budget of different species (e.g., Joshi *et al*. 1995; Nekaris 2001; Miard 2020).

Furthermore, it indicates that the species on Penang Island may be more used to human disturbance, which contradicts other studies where wild mammals would rather shift their activity times to later at night to avoid anthropogenetic activities (Gaynor *et al*. 2018). Species surveyed in our study, however, are arboreal and often well camouflaged and less visible compared to terrestrial species, which gives them a higher ability to evade human detections and a certain level of tolerance to human disturbance (Lowrey & Longshore 2017; Bista *et al*. 2022).

However, on Langkawi Island, we found that “canopy connectivity” was the most important factor in predicting species detection, indicating preferences for habitats with higher canopy cover percentage (Oliver *et al*. 2019; Cudney-Valenzuela *et al*. 2022), which is expected for arboreal mammals who are highly dependent on intact canopy cover for their movement and diet (Cudney-Valenzuela *et al*. 2022).

#### Species specific

Results for *Iomys horsfieldii* included most of the method-specific factor in the best model, which means that the method was appropriate to detect this species in all surveyed habitat types (Buckland *et al*. 2004; Guillera-Arroita 2017). This species is highly adaptable and can live in a wide range of habitats including degraded ones (Aplin & Lunde 2016), but we did detect them less along wide roads. They were also detected more just after sunset, but wind and humidity negatively affected their detection. Strong wind can significantly affect their ability to glide between trees (Ando & Shiraishi 1993; Lim 2007), Their detection success was positively correlated with lower humidity. Temperature and humidity are known to impact an animals’ thermoregulation (Paterson 1981) but how humidity itself impacts thermoregulation is not fully understood as most studies focus on temperature, which has less impact at night than during the day due to lower temperature fluctuation (Lopes & Bicca-Marques 2017). Humidity influences transpiration and water evaporation (Berglund 1998) and is expected to influence postural behaviour and microhabitat choice of mammals (Lopes & Bicca-Marques 2017).

*Galeopterus variegatus* is known as a rather elusive species (Lim & Ng 2010) but in this survey, it was the most common species sighted on both islands. *G. variegatus* can be found in mainland Peninsular Malaysia at all elevations and on the islands of Aur, Langkawi, Pangkor, Penang, Perhentian and Tioman, occurring in orchards, plantations, forests and woodlands (Dzulhelmi & Abdullah 2010; Medway 1969; Nasir 2013). Results for *G. variegatus* on Penang Island demonstrated that the study design works for this species as most of the factors under method-specific were included in the best model. This means that these animals were detected under most conditions tested by these variables (Zuur *et al*. 2009). They were detected along all path types but less detected on wide roads, and their behaviour showed an activity peak just after sunset, which is when they wake up to forage for food (Miard 2020). Their detection was higher on nights with lower cloud cover and temperature but higher wind speeds, which indicates that this species is more active during clear, colder, windier nights. Temperature correlates with anthropogenic disturbances, for example construction areas and cities have a higher temperature than surrounding forests, which could explain why detection rate was higher where temperature was lower as this may have correlated to less disturbed sites (Garvey *et al*. 2022).

Less cloud cover on the highly developed Penang Island could mean generally darker skies during clear nights as cloud cover enhances light pollution through reflection and diffusion (Linley *et al*. 2020). Moon illumination intensity affects animal movements and behaviour but also our ability to detect them, especially for well-camouflaged animals such as colugos (Rode-Margono & Nekaris 2014; Linley *et al*. 2020). This would mean that colugos are more active during darker nights. Higher wind speed seems to affect their movements positively. Wind can either highly disturb movements or help movements of a gliding species and their ability to glide and land on trees, depending on the body size and weight (Ando & Shiraishi 1993; Lim 2007). Colugos are considered big for a small mammal species weighing up to 2 kg, and wind may enhance their gliding distance and help with their airborne movements (Lim 2007).

In Langkawi, the site-specific factors were the best predictors for the detection of Sunda colugos indicating their occurrence in all studied sites in Langkawi (Zuur *et al* 2009). This could not be detected on Penang Island, likely due to the more intense anthropogenic disturbances and less available pristine habitats (Lim *et al*. 2013; Guillera-Arroita 2017). Relative to forest habitat they were more detected in villages and parks, but less in orchards and plantations. Colugos can adapt to different vegetation types including gardens, primary and secondary forests, rubber and coconut plantations, fruit orchards, mangrove swamps, lowlands and upland forests, tree plantations, lowland dipterocarp forests, and mountainous areas (Lim *et al*. 2013; Nasir 2013), however, not all habitat types can sustain large colugo populations (Lim *et al*. 2013).

Results for *Nycticebus coucang* were also different for both islands with disturbance being the most significant variable in predicting species detection on Langkawi Island, but on Penang Island only cloud, humidity and time after sunset had an impact on their detection. This could indicate some behavioural specificity in terms of activity times as the survey was always conducted during the same time frame, or a bias in the method, as higher humidity and cloud cover can negatively impact thermal detection using the FLIR (Main *et al*. 2012; Guillera-Arroita 2017). Slow lorises were detected more often just after sunset on Penang Island, but not on Langkawi Island. Due to more pristine forest habitat in Langkawi, slow lorises may come out to forest edges, where they are more easily detected, later at night compared to Penang, as they may spend their peak foraging time just after waking up inside the forest (Voskamp *et al*. 2014). The result for Langkawi Island indicates a preference for more pristine habitats, but when anthropogenic disturbance is high, slow lorises can adapt to disturbed areas inside agricultural landscapes, like on Penang Island (Voskamp *et al*. 2014). The forests in which *N. coucang* is normally sighted have a continuous substrate that allows these non-leaping, non-gliding arboreal mammals to move efficiently in the upper forest layers (Emmons & Gentry 1983). However, a study by Medway (1969) recorded that the distribution of *N. coucang* in Malaysia was widespread in plantations, forests, and mildly disturbed suburban gardens close to forests on the mainland and islands of Tioman, Pangkor and Penang. Slow lorises are known to walk on the ground, but it is not their preferred mode of locomotion, or even on powerlines and roof of houses, but this might be habitat dependent (Rode-Margono *et al*. 2014; Wiens 2002). In fact, some studies have reported slow lorises to thrive in villages and agricultural settings (Rode-Margono *et al*. 2014; Wiens 2002).

*Paradoxurus hermaphroditus* are disturbance-tolerant frugivorous mammals that disperse large seeds (Nakashima *et al*. 2010) and can survive in a broad range of habitats, including primary and secondary forest, urban areas, and cultivated land. Results for *P. hermaphroditus* were similar for both islands with the *time after sunset* best predicting their detection, which indicates a behavioural trait of the species (Joshi *et al*. 1995). However, they were detected more often just after sunset on Penang Island, but later at night on Langkawi Island, which may indicate hunting pressure (Lima & Dill 1990; Ferrari *et al*. 2009; Monterroso *et al. 2013*) *on Langkawi Island*, where the local population hunts them for food or to keep as pets (P. Miard, personal observation, 2020). On Langkawi Island, the *wind* speed was also included in the best predictor model, which could indicate a behavioural preference of civets for nights with less wind (Guillera-Arroita 2017).

Results for *P. petaurista* were also different on both islands, with predictors in the best model for Penang Island being *disturbance* and *distances to road*, both negatively impacting their detection. There is limited available habitat on island as larger flying squirrels prefer tall, mature trees in large old-stand vegetation patches and are found mostly at the canopy level (Barret 1985). According to Lee (1986), *P. petaurista*, a folivorous species, can be found at elevations of 300 m to 2,200 m in conifer and hardwood forests. They have also adapted to orchard plantations and secondary forests (Lee 1986).

On Langkawi Island, the best predictors for *P. petaurista* were *path type* and *time after sunset*, which both positively impacted their detection. This indicates habitat preferences and behavioural traits (Barret 1985; Lee 1986) as well as our ability to detect them up in the canopy as they were more sighted from open roads near forests than along dense forest trails. They were detected later at night, which can indicate their general behaviour of avoidance to human activities (Lima & Dill 1990; Ferrari e*t al*. 2009; Monterroso *et al. 2013*).

Overall, animal detection was mostly influenced by behavioural and habitat selection variables (Buckland *et al*. 2004; Guillera-Arroita 2017). Similar to another study, thermal imaging improved the study output by doubling the number of detected animals compared to spotlighting transect survey using white light (Underwood *et al*. 2022). Other method comparison studies, such as camera traps, driven transects and *ad hoc* records, have indeed shown that results for detecting nocturnal wildlife can be highly variable (Hart *et al*. 2022).

## RECOMMENDATIONS FOR NOCTURNAL MAMMAL RESEARCH

Although we could calculate encounter rates for each of the 17 detected species in this study, the method was unsuccessful to calculate the abundance, probably due to the generally lower detection probability of nocturnal mammals compared to diurnal mammals (Nekaris *et al*. 2008; Pereira *et al*. 2017; Rocha *et al*. 2021). Studying the behaviour of nocturnal mammals is generally more difficult than for diurnal wildlife, as they are more elusive and do not occur in larger social groups like many diurnal mammal species. Still, this study has contributed new important information on how these species may be affected by certain habitat variables, including anthropogenic disturbance, which ultimately can inform species-specific conservation actions.

Species have different behavioural patterns, and nocturnal mammals are active at different times of the night (Beier 2006; Halle & Stenseth 2012), which explains that the survey time can influence species detection, and this should be taken into consideration when designing similar or more species-specific research.

The following recommendations are made to further test and improve methods to study nocturnal mammals in the wild:

1. It is recommended to use at least 20 transects of 500 m length each with survey points every 100 m (total of 80 survey points) to study arboreal nocturnal mammals, especially if the aim is to record rare species. However, due to time and logistical constraints, this was not possible in this study.
2. Repeat the survey with the same specifications and a minimum of 80 survey points (for three repetitions, a minimum of 27 individual survey points is required, which corresponds to seven transects with 500 in length).
3. Conduct a similar survey in an area where all species and their abundances are known to estimate the efficiency of the method in terms of detection success of species and individuals.
4. One compulsory requirement for nocturnal mammal surveys is the use of red light instead of white light as it causes less bias in data collection due reduced flight response and harm to the nocturnal animals’ sensitive eyes, as they cannot see red light (Miard 2020). Red light improves detection rate with an increase of 45% for the detection of species and 46% for the detection of individuals compared to the use of white light (Miard 2020).

Standardised methods to monitor populations of many species are still lacking due to the facts that the basic ecology of many nocturnal mammals is widely understudies, although knowledge about their behavioural ecology is important for conservation purposes (Thompson 2004). Many studies have assessed the potential of tools, such as live trapping, camera trapping and thermal imaging for night surveys of wildlife (Green *et al*. 2020; Palmeirim *et al*. 2020). However, not many studies have assessed how to improve detection of nocturnal arboreal mammals by using traditional transect walks, maybe because new technologies are generally regarded as the better method (Green *et al*. 2020; Palmeirim *et al*. 2020). However, due to the high-cost factor for most new technologies used at night, many researchers may not have the resources to afford them, and therefore proper method improvement for transects surveys should be conducted.

## Figures and Tables

**Table 1:** Study sites description, with geographical coordinates, number of transects, habitat type, transect type, effort (km), transect gradient (m), number of nights surveyed, and disturbance of sites surveyed.

Island	Site	Coordinates	Number of transects	Habitat type	Transect type	Effort (km)	Transect gradient (m)	No. of nights surveyed	Disturbance (%)	Patch size (km^2^)
Penang	Ayer Hitam Dam (A)	5.398 N 100.263 E	15	Dipterocarp forest	Forest road	7.5	10–423	6	12.78	2.911
	Balik Pulau (BP)	5.365 N 100.240 E	15	Orchard	Farm trails	7.5	9–289	5	41.73	3.735
	Botanic Garden (BO)	5.440 N 100.289 E	9	Dipterocarp forest/Park	Road/Forest trail	4.5	29–260	5	29	0.717
	Bukit Gambir (BUG)	5.373 N 100.288 E	16	Orchard	Farm roads	8	36–346	10	19.66	1.500
	Bayan Lepas North (BN)	5.332 N 100.242 E	11	Orchard	Farm trails	5.5	43–361	5	26.58	2.298
	Bayan lepas South (BS)	5.306 N 100.247 E	19	Orchard	Farm trails	9.5	35–317	9	17.95	1.457
	Gertak Sanggul (G)	5.293 N 100.200 E	15	Orchard	Farm trails/Road	7.5	−26–224	5	15.25	5.588
	National Park (NP)	5.457 N 100.19 E	15	Dipterocarp forest	Forest trail	7.5	−6–144	6	0.47	4.382
	Penang Hill (PH)	5.421 N 100.265 E	16	Dipterocarp forest/Park	Road/Forest trail	8	631–782	7	5.73	2.351
	Taman Rimba	5.445 N 100.220 E	10	Dipterocarp forest/Park	Forest trails	5	28–237	11	3.39	0.722
	Tropical Spice (TS)	5.462 N 100.244 E	15	Dipterocarp forest	Road/Forest trail	7.5	0–122	9	12.53	3.608
	USM	5.356 N 100.300 E	15	City	Road	7.5	9–63	3	79.52	2.398
	Youth Park (YP)	5.432 N 100.291 E	16	Dipterocarp forest	Road/forest trail	8	22–334	8	17.65	1.583
Langkawi	Bukit Malut (BM)	6.299 N 99.791 E	15	Dipterocarp forests/Orchards	Forest trail/road	7.5	9–55	6	4.51	2.595
	Cable Car (CB)	6.379 N 99.672 E	10	Dipterocarp forests/Park	Forest trail/road	5	5–245	6	9.26	1.058
	ELS Datai (G)	6.429 N 99.685 E	15	Dipterocarp forests/Park	Forest trail	7.5	8–62	6	31.27	4.589
	Gua Maha (S)	6.341 N 99.852 E	15	Dipterocarp forests/Village	Village road	7.5	18–134	5	15.21	3.811
	Gunung Raya (GR)	6.387 N 99.802 E	15	Dipterocarp forests	Forest trail/road	7.5	11–604	5	1.48	3.995
	Kampung Kilim (K)	6.404 N 99.854 E	15	Dipterocarp forest/Plantation/Village	Village road	7.5	2–142	4	32.49	3.749
	Kedawang (LB)	6.309 N 99.758 E	16	Village/Plantation/Orchards	Village road	8	−1–162	5	27.97	4.093
	Kuah (P)	6.322 N 99.865 E	15	Dipterocarp forests/Village	Village road	7.5	6–241	6	38.74	4.285
	Lubok Setol (KT)	6.380 N 99.744 E	15	Dipterocarp forests/Village/Plantation	Village road/forest trail	7.5	−0.9–163	5	20.98	2.016
	Padang Gaong (PG)	6.341 N 99.800 E	15	Dipterocarp forests/Village	Village road	7.5	26–187	6	23.64	1.413
	Tanjung Rhu (TR)	6.437 N 99.807 E	15	Casuarina trees beach/Paddy field	Village road	7.5	6–19	4	21.62	5.768

**Table 2:** Factors used to model the General Linear Mixed Model for site-specific and method-specific of detection of nocturnal mammals on Penang and Langkawi islands.

Model	Factor	Description
Site-specific	Altitude (m)	Given by GPS
	Canopy connectivity	Visually assessed (scale 1: no connection; 2: at least two trees touching each other; 3: canopy fully closed)
	Canopy cover index (%)	Calculated in QGIS
	Distance to human settlements (in m)	Calculated with *Distance to nearest hub* tool of QGIS
	Distance to road (in m)	Calculated with *Distance to nearest hub* tool of QGIS
	Habitat patch size (km^2^)	Calculated in QGIS
	Habitat type	Classified as forest, orchard, plantation, village, city.
	Vegetation patch size (km^2^)	Calculated in QGIS
	Disturbance	Calculated in QGIS
Method-specific	Cloud cover	Visually assessed (scale 1: no clouds; 2: low to medium cloud cover; 3: heavy cover/overcast).
	Humidity (%)	Measured with a hygrometer
	Moon light (%)	Percentage of light (Phone application: *Phases of the Moon)*
	Path type	Classified as big road, village road, forest road, forest trail.
	Rain	Visually assessed (scale 1: no rain; 2: light to medium rainfall/drizzle; 3: heavy downpour).
	Temperature (°C)	Recorded with thermometer in the field
	Time of sighting after sunset	Calculated in Excel between time of sighting and sunset time.
	Wind	Visually assessed (scale 1: no wind; 2: light to medium windspeeds/breeze; 3: heavy windspeed/storm).

**Table 3:** Nocturnal non-volant mammal species sighted on Penang and Langkawi islands with their encounter rates.

Species	Penang Island		Langkawi Island	
	Number of sightings	Encounter rate ± SE (ind. km^−1^)	Number of sightings	Encounter rate ± SE (ind. km^−1^)
*Arctogalidia trivirgata*	n/a	n/a	1	0.01 ± 0.1
*Galeopterus variegatus*	122	1.30 ± 1.7	**131**	**1.65 ± 2.4**
*Hemigalus derbyanus*	1	0.01 ± 0.1	n/a	n/a
*Hylopetes spadiceus*	n/a	n/a	16	0.20 ± 0.7
*Hystrix brachyura*	n/a	n/a	1	0.01 ± 0.1
*Iomys horsfieldii* *	**134**	**1.43 ± 2.0**	n/a	n/a
*Lenothrix canus*	3	0.03 ± 0.1	n/a	n/a
*Nycticebus coucang*	11	0.12 ± 0.4	23	0.29 ± 0.6
*Lutrogale perspicillata*	n/a	n/a	8	0.10 ± 0.5
*Paguma larvata*	2	0.02 ± 0.1	1	0.01 ± 0.1
*Paradoxurus hermaphroditus*	38	0.41 ± 0.5	23	0.29 ± 0.4
*Petaurista petaurista*	12	0.13 ± 0.5	15	0.19 ± 0.6
*Prionailurus bengalensis*	1	0.01 ± 0.1	n/a	n/a
*Prionodon linsang*	n/a	n/a	1	0.01 ± 0.1
*Sus scrofa*	4	0.04 ± 0.3	4	0.05 ± 0.3
*Tragulus kanchil*	7	0.07 ± 0.2	4	0.05 ± 0.2
*Trichys fasciculata*	1	0.1	n/a	n/a

*Notes:*

* Detections mainly through animal calls (73% calls vs. 27% sightings).

n/a: not detected during the survey. The numbers in bold represent the highest values.

**Table 4:** Diversity of each survey site in Penang Island with biodiversity indexes and canopy cover index (CI) representing the percentage of area with low canopy cover per site.

Site	Number of sightings	Species richness (S)	Pielou’s evenness (J’)	Shannon-Wiener diversity index (H’)	Simpson diversity index (D)	Canopy cover index (CI) (%)
Ayer Hitam	37	4	0.7472	1.0358	0.6066	87.22
Balik Pulau	26	4	0.7018	0.9729	0.5631	58.27
Batu Feringghi	37	**7**	0.8049	1.5664	0.7432	87.47
Bayan Lepas North	**38**	4	0.6394	0.8864	0.5960	73.42
Bayan Lepas South	25	3	0.9456	1.0388	0.6533	82.05
Botanic Gardens	20	3	0.8083	0.8880	0.5947	70.98
Bukit Gambir	9	5	**0.9824**	**1.5811**	**0.8889**	80.34
Gertak Sanggul	30	4	0.7780	1.0785	0.6276	84.75
National Park	29	6	0.7414	1.3284	0.6921	**99.53**
Penang Hill	35	4	0.8843	1.2259	0.6840	94.27
Taman Rimba	33	**7**	0.5428	1.0562	0.5985	96.61
USM Campus	2	1	0	0.3342	0	20.48
Youth Park	10	3	0.8650	0.9503	0.6222	82.35

*Note:*

* The numbers in bold represent highest values.

**Table 5:** Diversity of each survey site in Langkawi Island with biodiversity indexes and Canopy cover index (CI).

Site	Number of sightings	Species richness (S)	Pielou’s evenness (J’)	Shannon diversity index (H’)	Simpson diversity index (D)	Canopy cover index (CI) (%)
Bukit Lembu	27	4	0.4726	0.6551	0.3333	72.03
Bukit Malut	24	3	0.6937	0.7621	0.4891	95.49
Cable car	28	**8**	0.6973	**1.4500**	0.7487	90.74
Golf Course	36	4	0.7948	1.1018	0.6381	68.73
Gunung Raya	11	4	0.8950	1.2407	0.7455	**98.52**
Kilim	4	3	**0.9464**	1.0397	**0.8333**	67.51
Kuala Teriang	19	4	0.6803	0.9430	0.5205	79.02
Padang Gaong	22	5	0.4873	0.7843	0.4675	76.36
Penarak	19	6	0.7733	1.3856	0.7018	61.26
Sungai Tarom	30	5	0.6375	1.0259	0.5333	84.79
Tanjung Rhu	2	1	0	0	0	78.38

*Note:*

* The numbers in bold represent highest values.

**Table 6:** Selection of the best model for the Generalised Linear Mixed Models (GLMM) of the factors affecting the detection of nocturnal mammals with the estimates for each variable. (Chosen from Delta AIC<2, bold numbers represent the best models).

Species	Location	Variables	df	Log-likelihood	AICc	ΔAICc	AICcwt
All species	Penang	**Time after sunset (−3.513)**	**2**	−**248.1**	**502.1333**	**0**	**0.517**
	Langkawi	**Canopy connectivity** (**0.691)**	**2**	−**491.9**	**989.8987**	**0**	**0.317**
		Canopy connectivity (0.672) + Distance settlement (−0.0005)	3	−491	989.9953	0.10	0.302
		Canopy connectivity (0.683) + Distance settlement (0.0001) + Altitude (−0.002)	4	−490.6	991.2417	1.34	0.162
*Galeopterus variegatus*	Penang	**Path type (Forest road: 16.699, Forest trail: 16.159, Village road: 14.956) + Cloud (**−**0.457) + Temperature (**−**0.038) + Wind (0.646) + Time after sunset (**−**1.025)**	**6**	−**260.6**	**539.2888**	**0**	**0.398**
		Path type (Forest road: 16.580, Forest trail: 16.080, Village road: 14.830) + Cloud (−0.478) + Temperature (−0.073) + Wind (0.6978) + Moon (−0.004) + Time after sunset (−1.035)	7	−260.0	540.1137	0.82	0.264
		Detection model (Forest road: 16.49, Forest trail: 16.04, Village road: 14.79, Cloud: −0.523, Temperature: 0.025, Moon: −0.004, Wind: 0.752, Humidity: 0.029, Time after sunset: −1.031)	8	−259.3	540.7395	1.45	0.193
	Langkawi	**Distribution model (Habitat type: Orchard:** −**0.581, Park: 0.260, Plantation:** −**0.628, Village: 0.998; Altitude:** −**0.003, Canopy connectivity: 0.831, Distance road: 0.00002, Distance settlement: 0.0004, Vegetation patch size: -0.364, Disturbance: 0.003)**	**8**	−**337.0**	**698.3025**	0	0.618
*Iomys horsfieldii*	Penang	**Path type (Forest road: 0.765, Forest trail: 1.616, Village road: 1.742) + Temperature (0.032) + Wind (**−**0.628) + Humidity (**−**0.051) + Time after Sunset (**−**1.205)**	**6**	−**226.7**	**471.6213**	**0**	**0.415**
		Trail type (Forest road: 0.833, Forest trail: 1.692, Village road 1.861) + Humidity (−0.045) + Time after sunset (−1.199)	4	−229.3	472.6887	1.07	0.243
*Nycticebus coucang*	Penang	**Cloud (0.950) + Humidity (**−**0.089) + Time after sunset (**−**0.915)**	**4**	−**29.9**	**69.90600**	**0**	**0.223**
		Time after sunset (−0.947) + Humidity (−0.088)	3	−31.0	70.10573	0.20	0.202
		Cloud (0.940) + Time after sunset (−0.85)	3	−31.4	70.81108	0.91	0.142
		Time after sunset (−0.870)	2	−32.5	70.92635	1.02	0.134
		Time after sunset (−0.878) + Wind (0.912)	3	−31.6	71.31129	1.41	0.111
		Cloud (0.874) + Wind (0.266) + Humidity (−0.078) + Time after sunset (−0.906)	5	−29.9	71.79502	1.89	0.087
	Langkawi	**Disturbance (**−**0.057)**	**2**	−**103.9**	**213.9122**	**0**	**0.350**
		Disturbance (−0.059) + Canopy connectivity (0.280)	3	−103.4	214.7975	0.89	0.225
*Paradoxurus hermaphroditus*	Penang	**Time after sunset (**−**0.8685)**	**2**	−**112.0**	229.9292	0	0.520
		Time after sunset (−0.871) + Temperature (−0.015)	3	−111.9	231.9281	1.99	0.192
	Langkawi	**Time after sunset (0.488) + Wind (**−**0.689)**	**3**	−**106.5**	**221.1157**	**0**	**0.165**
		Distance road (−0.002) + Distance habitation (0.0002) + Canopy connectivity (0.540)	4	−105.5	221.1403	0.02	0.163
		Distance road (−0.002)	2	−107.6	221.1651	0.05	0.161
		Time after sunset (0.5318)	2	−107.7	221.4350	0.32	0.140
		Distance habitation (−0.00008)	2	−108.2	222.4428	1.33	0.085
		Time after sunset (0.474) + Wind (−0.530) + Humidity (0.021)	4	−106.2	222.5161	1.40	0.082
*Petaurista petaurista*	Penang	**Disturbance (**−**0.234) + Distance road** (−**0.005)**	**3**	−**29.8**	**67.62871**	**0**	0.281
		Disturbance (−0.220) + Canopy connectivity (0.491) + Distance road (−0.006)	4	−29.5	69.02872	1.40	0.140
		Altitude (0.001) + Disturbance (−0.214) + Distance road (−0.004)	4	−29.5	69.05982	1.43	0.137
		Disturbance (−0.219) + Distance road (−0.004) + Distance habitation (0.0003)	4	−29.7	69.45906	1.83	0.113
	Langkawi	**Path type (Forest road: 17.851, Forest trail: 15.902, Village road: 16.056) + Time after sunset (0.621)**	**3**	−**62.6**	**137.2607**	**0**	**0.432**
		Path type (Forest road: 17.930, Forest trail:16.068, Village road 16.167) + Time after sunset (0.562) + Wind (−0.612)	4	−61.9	137.9935	0.73	0.299

## APPENDIX

Nocturnal mammal sighting accumulation curves per number of sites surveyed for Penang Island (left) and Langkawi Island (right) produced by 100 random reorderings (RStudio 2017, *Vegan* package*)*. The different lines represent different species.[Fig f2-tlsr_35-1-49]
